# Doctors’ smoking control knowledge, attitudes and practices: a cross-sectional study conducted in Shandong Province, China

**DOI:** 10.1186/s12889-020-10076-x

**Published:** 2021-01-07

**Authors:** Qiang Wang, Xueli Zhang, Zengwu Wang, Shangang Feng, Yang Li, Chuanfeng Zhang, Chunping Wang

**Affiliations:** 1 Department of Epidemiology, Weifang Medical University, Weifang, Shandong China; 2 Department of Histology and Embryology, Weifang Medical University, Weifang, Shandong China; 3 Weifang people’s hospital, Weifang, Shandong China; 4 School of Public health and Management, Weifang Medical University, Weifang, Shandong China; 5 Department of Environmental Health, Weifang Medical University, No. 7166 Baotong west street, Weifang, 261053 Shandong People’s Republic of China

**Keywords:** Smoking control, Knowledge, Attitude, Practice

## Abstract

**Background:**

Doctors play an important role in smoking control. This study aimed to assess doctors’ smoking control knowledge, attitudes and practices to help doctors raise awareness of smoking control assistance.

**Methods:**

This cross-sectional study recruited 1046 doctors from Shandong Province, China, by using multistage sampling. Participants’ information was collected by questionnaire. Pearson’s χ^2^ test and Fisher’s exact probability method were used to compare the distributions of categorical variables between/among groups.

**Results:**

Among the participants, 14.7% were current smokers. Approximately 50.3% of participants had heard of smoking cessation drugs and 59.2% of participants thought that low-tar and low-nicotine cigarettes were as harmful to health as common cigarettes. Approximately 98.2 and 60.9% of participants agreed that smoking was related to lung cancer and male sexual dysfunction, respectively. Although 72.0% of participants believed that doctors should actively provide smoking cessation assistance, only 58.1% of participants considered that doctors should be responsible for providing smoking cessation assistance. Similarly, 85.2% of participants often asked about the smoking history of patients or their family members, while only 4.9% of participants had prescribed smoking cessation drugs for patients. Pediatricians had a higher proportion of “Agree” responses to the assessment items than doctors in other departments.

**Conclusions:**

The results showed that doctors in Shandong Province did not have sufficient knowledge of smoking control. Slightly more than half of doctors thought that providing smoking cessation assistance was their responsibility. Only a few participants had prescribed smoking cessation drugs.

## Background

Smoking is a preventable risk factor for many noncommunicable diseases [1]. Many interventions have been implemented to prevent smoking. However, the prevalence of smoking is still high, and the problems resulting from smoking are still serious. Worldwide, approximately 20% of people aged ≥15 years were current smokers in 2015 [2]. Tobacco is responsible for seven million deaths annually [3]. In China, there are up to 300 million smokers [4]. According to the report of the Chinese Center for Disease Control and Prevention, 50.5% of males aged ≥15 years in China were smokers in 2018 [5]. In addition, the rate of secondhand smoke exposure among nonsmoking people was 68.1% [5]. As a result, there were up to 1 million deaths related to smoking in China [6]. Thus, it is urgent to help smokers quit smoking, especially in China.

Counseling by doctors combined with pharmacotherapy is an effective smoking cessation intervention [7]. Simple and brief counseling by a doctor increased the probability of quitting smoking by 66% [8]. Smokers who were provided counseling and medication had a twofold higher likelihood of quitting smoking successfully than smokers who did not receive counseling and medication [9]. Doctors are regarded as role models for healthy lifestyles by patients [10]. In addition, doctors’ counseling is more likely to be accepted by smoking patients due to doctors’ specialized knowledge. Thus, doctors play a crucial role in providing counseling about smoking cessation [11]. Obviously, many studies have also proposed that doctors should theoretically provide counseling about smoking cessation for smokers in daily work [12]. However, doctors’ responses to smoking control shown in previous studies are not satisfactory. A cross-sectional study conducted in China demonstrated that the harms of smoking were not well known by all doctors [10]. A cross-sectional study carried out in Egypt in 2011 showed that more than 50% of doctors admitted that they had poor knowledge of smoking cessation interventions [9]. A qualitative study implemented in Armenia in 2015 reported that some doctors did not consider smoking cessation counseling to be their responsibility [7]. The 5As method (Ask: ask all patients about tobacco use; Advise: advise all tobacco users to quit smoking; Assess: assess the willingness to quit smoking; Assist: assist with quitting; Arrange: arrange follow-up) refers to one common strategy to address tobacco use and nicotine dependence [12]. A systematic literature review conducted in 2016 showed that 65% of doctors asked about their patients’ smoking behavior, and 63% advised their patients to quit smoking [13]. In addition, this review reported that approximately 36, 44 and 22% of doctors assessed, assisted and arranged follow-up with their patients, respectively [13]. As the data demonstrated, the doctors’ factual responses to smoking control do not match the doctors’ expected responses. Thus, doctors’ factual performance in smoking control should be reassessed and enhanced.

As China produces the largest number of cigarettes and has the highest number of smokers and tobacco-related deaths [14], it is necessary to implement a smoke-free policy in China and help smokers quit smoking. The medical doctors’ role in smoking control is particularly important. Shandong Province has the second largest population in China [15]. However, few studies have investigated the performance of doctors in smoking control in Shandong Province. In this study, we aimed to assess the factual representations of doctors registered in Shandong Province in smoking control based on their knowledge, attitudes and practices about smoking control, which may be able to fill the previous research gap and raise doctors’ awareness of smoking control assistance.

## Methods

### Definitions

Smokers were defined as people who had smoked 100 or more cigarettes (or the equivalent amount of tobacco) during their lifetime [16]. Current smokers were defined as smokers who smoked any tobacco product at the time of the survey. Ex-smokers were defined as smokers who had not smoked at all in the last 3 months at the time of the survey. Never-smokers were defined as people who had smoked no more than 100 cigarettes (or the equivalent amount of tobacco) during their lifetime. Nonsmokers were defined as ex-smokers and never-smokers in this study.

### Sample size

The formula used to calculate the sample size was as follows: *N* = 400 × *q*÷*p* [17]. In this formula, *N* is the sample size and *p* is the incident rate of some event. *q* = 1-*p*. A previous study conducted in China showed that the prevalence of current smoking among doctors was 28.4% [18]. Therefore, we entered *p* = 28.4% into the formula and calculated the sample size to be 1008. The last step of the sampling under study was cluster sampling. After the cluster sampling, the number of doctors included in this study was 1050. Therefore, the required sample size was changed to 1050.

### Participants

A cross-sectional study was conducted in Shandong Province, China, from July to December, 2018. Multistage sampling was carried out to select participants. Firstly, 17 cities of Shandong Province were divided into two groups according to social, cultural and geographical conditions. Secondly, one city was randomly sampled from each group as the sample city. Weifang and Liaocheng were selected. Thirdly, one 3-A-Class comprehensive hospital in Weifang and two 3-A-Class comprehensive hospitals in Liaocheng were randomly sampled as the sample hospitals, including Weifang People’s Hospital, Liaocheng People’s Hospital and Liaocheng Traditional Chinese Medicine Hospital. Fourthly, cluster sampling was conducted separately in each hospital to randomly sample departments. All doctors within the sampled departments were treated as participants and interviewed by the questionnaire used in the current study. This study was approved by the Ethics Review Committee of Weifang Medical University, and informed consent was obtained from all participants.

A total of 1050 doctors were included in this study, and 1046 valid questionnaires were retrieved (valid response rate: 99.6%). Data from the 1046 valid questionnaires were analyzed.

### Questionnaire

Every participant attended a face-to-face interview with well-trained investigators and completed a questionnaire designed based on the *Questionnaire of Key Population’s Smoking Behavior* published by the Chinese Center for Disease Control and Prevention [19]. The current questionnaire included the participants’ sociodemographic data, smoking status and knowledge, attitudes and practices in smoking control.

The questionnaire used in the present study had acceptable levels of reliability and validity. The internal consistency reliability test showed that the Cronbach’s alpha coefficient was 0.85. The construct validity test showed that the Kaiser-Meyer-Olkin was 0.84, and Bartlett’s test of sphericity produced a result of χ^2^ value = 8908.7 (*P* < 0.01).

### Statistical analysis

Pearson’s χ^2^ test and Fisher’s exact probability method were used to compare the distribution of the categorical variables between/among groups. Statistical analyses were performed by Stata version 13.1. All reported probabilities (*P* values) were two sided. *P* < 0.05 was considered statistically significant.

## Results

### Demographic characteristics of participants

Table 1 shows the demographic characteristics of the participants. Among the participants, 61.2% were male and 38.8% were female. Approximately 46.9% of the participants were aged between 31 and 40 years. Physicians, surgeons and pediatricians accounted for 32.7, 24.8 and 10.8% of the participants, respectively. Among the participants, 14.7% were current smokers.

**Table 1:** Demographic characteristics of participants

Variable	Subgroup	Frequency	Percentage (%)
Hospital	Weifang People’s Hospital	344	32.89
	Liaocheng People’s Hospital	624	59.66
	Liaocheng Traditional Chinese Medicine Hospital	78	7.46
Sex	1.male	640	61.19
	2.female	406	38.81
Age(years)	≤30	240	22.94
	≤40	491	46.94
	≤50	225	21.51
	> 50	90	8.60
Education	Bachelor’s degree or below	327	31.26
	Graduate degree	719	68.74
Professional title	No professional title	90	8.60
	Primary professional title	243	23.23
	Middle professional title	290	27.72
	Deputy senior professional title	262	25.05
	Senior professional title	161	15.39
Department	Physician	342	32.70
	Surgeon	259	24.76
	Obstetrics and gynecology	78	7.46
	Pediatrics	113	10.80
	Other departments	254	24.28
Smoking status	Smoker	154	14.72
	Nonsmoker	892	85.28

### Participants’ knowledge of smoking

The participants’ knowledge of smoking is summarized in Table 2. Among the participants, 81.4% agreed that smoking addiction is a cluster of cognitive, behavioral and physiological symptoms. In addition, 50.3% of the participants had heard that there are smoking cessation drugs. Approximately 46.9% of the participants responded to the question “Filter tips can decrease the harm from smoking” with “Agree”. Approximately 59.2% of the participants answered the question “Low-tar and low-nicotine cigarettes are as harmful to health as common cigarettes” with “Agree”. The rates of responses to the above four items with “Agree” or “Yes” were significantly different among the age groups, professional title groups and department groups, as well as between smoking status groups. Among the five doctor department groups, pediatricians and surgeons presented the highest and lowest proportions of responses to the above four items with “Agree”, respectively.

**Table 2:** Participants’ cognition of smoking

Variables		Smoking addiction is a cluster of cognitive, behavioral and physiological symptoms			Smoking cessation drugs			Filter tips can decrease the harm from smoking			Low-tar and low-nicotine cigarettes are as harmful to health as common cigarettes		
		Agree	Do not agree	*P*	Have heard	Do not know	*P*	Agree	Do not agree	*P*	Agree	Do not agree	*P*
Total number		851, 81.36	195, 18.64		526, 50.29	520, 49.71		491, 46.94	555, 53.06		619, 59.18	427, 40.82	
Sex	1	519, 81.09	121, 18.91	0.78	315, 49.22	325, 50.78	0.39	280, 43.75	360, 56.25	< 0.01	355, 55.47	285, 44.53	< 0.01
	2	332, 81.77	195, 18.64		211, 51.97	195, 48.03		211, 51.97	195, 48.03		264, 65.02	142, 34.98	
Age (years)	≤30	186, 77.50	54, 22.50	< 0.01	98, 40.83	142, 59.17	< 0.01	92, 38.33	148, 61.67	< 0.01	129, 53.75	111, 46.25	< 0.01
	≤40	390, 79.43	101, 20.57		238, 48.47	253, 51.53		199, 40.53	292, 59.47		276, 56.21	215, 43.79	
	≤50	189, 84.00	36, 16.00		126, 56.00	99, 44.00		130, 57.78	95, 42.22		142, 63.11	83, 36.89	
	> 50	86, 95.56	4, 4.44		64, 71.11	26, 28.89		70, 77.78	20, 22.22		72, 80.00	18, 20.00	
Education	1	257, 78.59	70, 21.41	0.12	136, 41.59	191, 58.41	< 0.01	138, 42.20	189, 57.80	0.04	187, 57.19	140, 42.81	0.38
	2	594, 82.61	125, 17.39		390, 54.24	329, 45.76		353, 49.10	366, 50.90		432, 60.08	287, 39.92	
Professional title	1	58, 64.44	32, 35.56	< 0.01	30, 33.33	60, 66.67	< 0.01	20, 22.22	70, 77.78	< 0.01	35, 38.89	55, 61.11	< 0.01
	2	192, 79.01	51, 20.99		103, 42.39	140, 57.61		83, 34.16	160, 65.84		112, 46.09	131, 53.91	
	3	246, 84.83	44, 15.17		144, 49.66	146, 50.34		132, 45.52	158, 54.48		181, 62.41	109, 37.59	
	4	213, 81.30	49, 18.70		145, 55.34	117, 44.66		156, 59.54	106, 40.46		177, 67.56	85, 32.44	
	5	142, 88.20	19, 11.80		104, 64.60	57, 35.40		100, 62.11	61, 37.89		114, 70.81	47, 29.19	
Department	1	277, 80.99	65, 19.01	< 0.01	150, 43.86	192, 56.14	< 0.01	102, 29.82	240, 70.18	< 0.01	161, 47.08	181, 52.92	< 0.01
	2	186, 71.81	73, 28.19		88, 33.98	171, 66.02		89, 34.36	170, 65.64		121, 46.72	138, 53.28	
	3	67, 85.90	11, 14.10		39, 50.00	39, 50.00		39, 50.00	39, 50.00		59, 75.64	19, 24.36	
	4	112, 99.12	1, 0.88		100, 88.50	13, 11.50		107, 94.69	6, 5.31		105, 92.92	8, 7.08	
	5	209, 82.28	45, 17.72		149, 58.66	105, 41.34		154, 60.63	100, 39.37		173, 68.11	81, 31.89	
Smoking status	1	115, 74.68	39, 25.32	0.02	65, 42.21	89, 57.79	0.03	42, 27.27	112, 72.73	< 0.01	67, 43.51	87, 56.49	< 0.01
	2	736, 82.51	156, 17.49		461, 51.68	431, 48.32		449, 50.34	443, 49.66		552, 61.88	340, 38.12	

Data are presented as frequency and percentage. Statistical analysis method: Pearson’s χ^2^ test. Sex: 1: male, 2: female; Education: 1: bachelor’s degree or below, 2: graduate degree; Professional title: 1: no professional title, 2: primary professional title, 3: middle professional title, 4: deputy senior professional title, 5: senior professional title; Department: 1: physician, 2: surgeon, 3: obstetrics and gynecology, 4: pediatrics; 5: other departments; Smoking status: 1: current smokers, 2: nonsmokers

### Participants’ knowledge of smoking-related diseases

Participants’ knowledge of smoking-related diseases is shown in Table 3. The participants who agreed that “smoking is related to lung cancer” and “smoking is related to coronary disease” accounted for 98.2 and 92.8% of the participants, respectively. The percentages of participants who responded to the questions “Smoking is related to osteoporosis” and “Smoking is related to male sexual dysfunction” with “Agree” were 56.2 and 60.9%, respectively. The rates of responses to the above four items with “Agree” were significantly different among the five department groups. In addition, the pediatrician group had the highest proportion of answers to three questions (“Smoking is related to coronary disease”, “Smoking is related to osteoporosis” and “Smoking is related to male sexual dysfunction”) of “Agree” among the five doctor department groups.

**Table 3:** Participants’ cognition of smoking-related diseases

Variables		Smoking is related to lung cancer			Smoking is related to coronary disease			Smoking is related to osteoporosis			Smoking is related to male sexual dysfunction		
		Agree	Do not agree	*P*	Agree	Do not agree	*P*	Agree	Do not agree	*P*	Agree	Do not agree	*P*
Total number		1027, 98.18	19, 1.82		971, 92.83	75, 7.17		588, 56.21	458, 43.79		637, 60.90	409, 39.10	
Sex	1	625, 97.66	15, 2.34	0.11	600, 93.75	40, 6.25	0.15	352, 55.00	288, 45.00	0.32	389, 60.78	251, 39.22	0.92
	2	402, 99.01	4, 0.99		371, 91.38	35, 8.62		236, 58.13	170, 41.87		248, 61.08	158, 38.92	
Age (years)	≤30	235, 97.92	5, 2.08	0.69^*^	216, 90.00	24, 10.00	0.03	118, 49.17	122, 50.83	< 0.01	132, 55.00	108, 45.00	< 0.01
	≤40	480, 97.76	11, 2.24		453, 92.26	38, 7.74		253, 51.53	238, 48.47		279, 56.82	212, 43.18	
	≤50	223, 99.11	2, 0.89		213, 94.67	12, 5.33		142, 63.11	83, 36.89		152, 67.56	73, 32.44	
	> 50	89, 98.89	1, 1.11		89, 98.89	1, 1.11		75, 83.33	15, 16.67		74, 82.22	16, 17.78	
Education	1	318, 97.25	9, 2.75	0.13	293, 89.60	34, 10.40	< 0.01	173, 52.91	154, 47.09	0.15	185, 56.57	142, 43.43	0.06
	2	709, 98.61	10, 1.39		678, 94.30	41, 5.70		415, 57.72	304, 42.28		452, 62.87	267, 37.13	
Professional title	1	88, 97.78	2, 2.22	0.65^*^	75, 83.33	15, 16.67	0.01	34, 37.78	56, 62.22	< 0.01	39, 43.33	51, 56.67	< 0.01
	2	240, 98.77	3, 1.23		226, 93.00	17, 7.00		111, 45.68	132, 54.32		130, 53.50	113, 46.50	
	3	283, 97.59	7, 2.41		270, 93.10	20, 6.90		176, 60.69	114, 39.31		187, 64.48	103, 35.52	
	4	259, 98.85	3, 1.15		246, 93.89	16, 6.11		163, 62.21	99, 37.79		167, 63.74	95, 36.26	
	5	157, 97.52	4, 2.48		154, 95.65	7, 4.35		104, 64.60	57, 35.40		114, 70.81	47, 29.19	
Department	1	337, 98.54	5, 1.46	0.03^*^	309, 90.35	33, 9.65	0.04	150, 43.86	192, 56.14	< 0.01	171, 50.00	171, 50.00	< 0.01
	2	251, 96.91	8, 3.09		237, 91.51	22, 8.49		108, 41.70	151, 58.30		135, 52.12	124, 47.88	
	3	74, 94.87	4, 5.13		74, 94.87	4, 5.13		45, 57.69	33, 42.31		55, 70.51	23, 29.49	
	4	112, 99.12	1, 0.88		111, 98.23	2, 1.77		109, 96.46	4, 3.54		104, 92.04	9, 7.96	
	5	253, 99.61	1, 0.39		240, 94.49	14, 5.51		176, 69.29	78, 30.71		172, 67.72	82, 32.28	
Smoking status	1	151, 98.05	3, 1.95	0.75^*^	143, 92.86	11, 7.14	0.99	72, 46.75	82, 53.25	0.01	88, 57.14	66, 42.86	0.30
	2	876, 98.21	16, 1.79		828, 92.83	64, 7.17		516, 57.85	376, 42.15		549, 61.55	343, 38.45	

Data are presented as frequency and percentage. Statistical analysis method: * is Fisher’s exact probability method and others are Pearson’s χ^2^ test. Sex: 1: male, 2: female; Education: 1: bachelor’s degree or below, 2: graduate degree; Professional title: 1: no professional title, 2: primary professional title, 3: middle professional title, 4: deputy senior professional title, 5: senior professional title; Department: 1: physician, 2: surgeon, 3: obstetrics and gynecology, 4: pediatrics; 5: other departments; Smoking status: 1: current smokers, 2: nonsmokers

### Participants’ attitudes towards smoking control

Participants’ attitudes towards smoking control are shown in Table 4. Among the participants, 81.9% agreed that doctors should be the role model of nonsmoking; 69.0% answered the question “The doctor is the best role to persuade smokers to quit” with “Agree”; 72.0% responded to the question “Doctors should provide smoking cessation assistance actively” with “Agree”; and 58.1% agreed that “Provision of smoking cessation assistance is the doctor’s responsibility”. The rates of responses to the above four items with “Agree” were significantly different among the age groups, professional title groups and department groups, as well as between smoking status groups. Among the five doctor department groups, pediatricians and surgeons presented the highest and lowest proportions of responses to the above four items with “Agree”, respectively.

**Table 4:** Participants’ attitudes about smoking

Variables		Doctor should be the role model of nonsmoking			Doctor is the best role to persuade smokers to quit			Doctor should provide smoking cessation assistance actively			Provision of smoking cessation assistance is doctor’s responsibility		
		Agree	Do not agree	*P*	Agree	Do not agree	*P*	Agree	Do not agree	*P*	Agree	Do not agree	*P*
Total number		857, 81.93	189, 18.07		722, 69.02	324, 30.98		753, 71.99	293, 28.01		608, 58.13	438, 41.87	
Sex	1	510, 79.69	130, 20.31	0.02	427, 66.72	213, 33.28	0.04	438, 68.44	202, 31.56	< 0.01	362, 56.56	278, 43.44	0.20
	2	347, 85.47	59, 14.53		295, 72.66	111, 27.34		315, 77.59	91, 22.41		246, 60.59	160, 39.41	
Age (years)	≤30	186, 77.50	54, 22.50	< 0.01	150, 62.50	90, 37.50	< 0.01	161, 67.08	79, 32.92	< 0.01	127, 52.92	113, 47.08	< 0.01
	≤40	397, 80.86	94, 19.14		318, 64.77	173, 35.23		336, 68.43	155, 31.57		257, 52.34	234, 47.66	
	≤50	188, 83.56	37, 16.44		171, 76.00	54, 24.00		172, 76.44	53, 23.56		149, 66.22	76, 33.78	
	> 50	86, 95.56	4, 4.44		83, 92.22	7, 7.78		84, 93.33	6, 6.67		75, 83.33	15, 16.67	
Education	1	266, 81.35	61, 18.65	0.74	201, 61.47	126, 38.53	< 0.01	230, 70.34	97, 29.66	0.42	175, 53.52	152, 46.48	0.04
	2	591, 82.20	128, 17.80		521, 72.46	198, 27.54		523, 72.74	196, 27.26		433, 60.22	286, 39.78	
Professional title	1	59, 65.56	31, 34.44	< 0.01	37, 41.11	53, 58.89	< 0.01	49, 54.44	41, 45.56	< 0.01	26, 28.89	64, 71.11	< 0.01
	2	189, 77.78	54, 22.22		154, 63.37	89, 36.63		144, 59.26	99, 40.74		127, 52.26	116, 47.74	
	3	241, 83.10	49, 16.90		197, 67.93	93, 32.07		219, 75.52	71, 24.48		179, 61.72	111, 38.28	
	4	223, 85.11	39, 14.89		201, 76.72	61, 23.28		204, 77.86	58, 22.14		160, 61.07	102, 38.93	
	5	145, 90.06	16, 9.94		133, 82.61	28, 17.39		137, 85.09	24, 14.91		116, 72.05	45, 27.95	
Department	1	271, 79.24	71, 20.76	< 0.01	221, 64.62	121, 35.38	< 0.01	221, 64.62	121, 35.38	< 0.01^*^	171, 50.00	171, 50.00	< 0.01
	2	198, 76.45	61, 23.55		155, 59.85	104, 40.15		153, 59.07	106, 40.93		124, 47.88	135, 52.12	
	3	74, 94.87	4, 5.13		74, 94.87	4, 5.13		71, 91.03	7, 8.97		41, 52.56	37, 47.44	
	4	113, 100.00	0, 0.00		111, 98.23	2, 1.77		113, 100.00	0, 0.00		102, 90.27	11, 9.73	
	5	201, 79.13	53, 20.87		161, 63.39	93, 36.61		195, 76.77	59, 23.23		170, 66.93	84, 33.07	
Smoking status	1	99, 64.29	55, 35.71	< 0.01	82, 53.25	72, 46.75	< 0.01	84, 54.55	70, 45.45	< 0.01	64, 41.56	90, 58.44	< 0.01
	2	758, 84.98	134, 15.02		640, 71.75	252, 28.25		669, 75.00	223, 25.00		544, 60.99	348, 39.01	

Data are presented as frequency and percentage. Statistical analysis method: * is Fisher’s exact probability method and others are Pearson’s χ^2^ test. Sex: 1: male, 2: female; Education: 1: bachelor’s degree or below, 2: graduate degree; Professional title: 1: no professional title, 2: primary professional title, 3: middle professional title, 4: deputy senior professional title, 5: senior professional title; Department: 1: physician, 2: surgeon, 3: obstetrics and gynecology, 4: pediatrics; 5: other departments; Smoking status: 1: current smokers, 2: nonsmokers

### Participants’ smoking control practices

Participants’ smoking control practices are summarized in Table 5. Among the participants, 85.2% often asked actively about the smoking history of patients or the patient’s family members. Approximately 67.3% of participants would often educate the patients about the harms of smoking and persuade the patients to quit smoking if the patients had the habit of smoking. Only 4.9% of the participants had prescribed smoking cessation drugs for patients. There were significant differences in the above three items among the professional title groups and department groups. Pediatricians presented the highest proportions of participants who often asked about the smoking history of the patients or the patient’s family members, educated the patients about the harm of smoking, persuaded the patients to quit smoking, and had prescribed smoking cessation drugs for patients.

**Table 5:** Participants’ actions about smoking

Variables		Ask about the smoking history of patients or the patients’ family members actively				Whether or not to educate the patients about the harm of smoking and persuade the patients to quit smoking if the patients have the habit of smoking				Have prescribed smoking cessation drug for patient		
		Often	When smoking is related to disease	Rarely	*P*	Often	Sometimes	Never	*P*	Yes	No	*P*
Total number		891, 85.18	91, 8.70	64, 6.12		704, 67.30	323, 30.88	19, 1.82		51, 4.88	995, 95.12	
Sex	1	556, 86.88	53, 8.28	31, 4.84	0.07	427, 66.72	196, 30.63	17, 2.66	0.04	34, 5.31	606, 94.69	0.41
	2	335, 82.51	38, 9.36	33, 8.13		277, 68.23	127, 31.28	2, 0.49		17, 4.19	389, 95.81	
Age (years)	≤30	204, 85.00	14, 5.83	22, 9.17	0.06	154, 64.17	79, 32.92	7, 2.92	< 0.01^*^	6, 2.50	234, 97.50	< 0.01^*^
	≤40	421, 85.74	41, 8.35	29, 5.91		309, 62.93	171, 34.83	11, 2.24		18, 3.67	473, 96.33	
	≤50	191, 84.89	27, 12.00	7, 3.11		162, 72.00	62, 27.56	1, 0.44		10, 4.44	215, 95.56	
	> 50	75, 83.33	9, 10.00	6, 6.67		79, 87.78	11, 12.22	0, 0.00		17, 18.89	73, 81.11	
Education	1	259, 79.20	37, 11.31	31, 9.48	< 0.01	212, 64.83	106, 32.42	9, 2.75	0.21	19, 5.81	308, 94.19	0.34
	2	632, 87.90	54, 7.51	33, 4.59		492, 68.43	217, 30.18	10, 1.39		32, 4.45	687, 95.55	
Professional title	1	82, 91.11	1, 1.11	7, 7.78	< 0.01	48, 53.33	38, 42.22	4, 4.44	< 0.01^*^	3, 3.33	87, 96.67	0.01^*^
	2	212, 87.24	12, 4.94	19, 7.82		150, 61.73	87, 35.80	6, 2.47		5, 2.06	238, 97.94	
	3	248, 85.52	27, 9.31	15, 5.17		196, 67.59	91, 31.38	3, 1.03		13, 4.48	277, 95.52	
	4	224, 85.50	22, 8.40	16, 6.11		182, 69.47	74, 28.24	6, 2.29		14, 5.34	248, 94.66	
	5	125, 77.64	29, 18.01	7, 4.35		128, 79.50	33, 20.50	0, 0.00		16, 9.94	145, 90.06	
Department	1	305, 89.18	25, 7.31	12, 3.51	< 0.01	247, 72.22	94, 27.49	1, 0.29	< 0.01^*^	11, 3.22	331, 96.78	< 0.01^*^
	2	218, 84.17	25, 9.65	16, 6.18		143, 55.21	104, 40.15	12, 4.63		9, 3.47	250, 96.53	
	3	53, 67.95	12, 15.38	13, 16.67		61, 78.21	17, 21.79	0, 0.00		1, 1.28	77, 98.72	
	4	102, 90.27	4, 3.54	7, 6.19		102, 90.27	11, 9.73	0, 0.00		11, 9.73	102, 90.27	
	5	213, 83.86	25, 9.84	16, 6.30		151, 59.45	97, 38.19	6, 2.36		19, 7.48	235, 92.52	
Smoking status	1	137, 88.96	9, 5.84	8, 5.19	0.33	76, 49.35	71, 46.10	7, 4.55	< 0.01^*^	11, 7.14	143, 92.86	0.16
	2	754, 84.53	82, 9.19	56, 6.28		628, 70.40	252, 28.25	12, 1.35		40, 4.48	852, 95.12	

Data are presented as frequency and percentage. Statistical analysis method: * is Fisher’s exact probability method and others are Pearson’s χ^2^ test. Sex: 1: male, 2: female; Education: 1: bachelor’s degree or below, 2: graduate degree; Professional title: 1: no professional title, 2: primary professional title, 3: middle professional title, 4: deputy senior professional title, 5: senior professional title; Department: 1: physician, 2: surgeon, 3: obstetrics and gynecology, 4: pediatrics; 5: other departments; Smoking status: 1: current smokers, 2: nonsmokers

## Discussion

In the current study, participants did not have sufficient (response rate for “Agree”: 45–60%) common knowledge of smoking-related harms (such as filter tips and low-tar and low-nicotine cigarettes). In contrast, participants had better (response rate for “Agree”: up to 80%) specific knowledge of the smoking-related harms that were associated with medical conditions (for example, “smoking addiction is a cluster of cognitive, behavioral and physiological symptoms”). The possible reason for this difference might be related to doctors’ educational experience. Specific knowledge of the smoking-related harms associated with medical conditions can be learned in medical universities but the most common knowledge of smoking-related harms is learned in special training on smoking control. However, most participants did not receive special training on smoking control, which suggested a shortage of special training on smoking control for Chinese doctors and other countries’ doctors [20–22]. As a result, doctors had little chance to learn the common knowledge of smoking-related harm. In conclusion, special training on smoking control is needed for doctors.

Participants were knowledgeable about the association between smoking and lung disease and coronary disease. However, they were not familiar with other uncommon smoking-related diseases (such as osteoporosis and male sexual dysfunction). This result was similar to a previous Chinese study [10], supporting that knowledge of the harm from smoking was still insufficient among Chinese doctors. Knowledge determines attitude and action. A previous study showed that scientific knowledge about smoking control was important for doctors to contribute to smoking control [10]. Therefore, doctors need more special training on smoking control.

In this study, most participants (approximately 80%) realized their key role in smoking control, which was similar to the results of previous studies conducted in China and other countries [7, 23, 24]. However, approximately 60% of participants believed that providing smoking cessation assistance was their responsibility, which was also consistent with the findings of previous studies [7, 20]. There were some possible explanations for the difference between the responses of the two items. Firstly, although the WHO states that every health professional is responsible for asking about patients’ tobacco use, assessing patients’ willingness to quit smoking, advising patients to quit smoking, and further referring and arranging patients to participate in smoking cessation plans [25], doctors have not been required to provide smoking cessation assistance in China [26]. Secondly, most Chinese doctors thought that they were too busy to provide smoking cessation counseling [26], which also occurred in other countries [20].

The 5As method refers to 5 different counseling strategies for smoking cessation, including “Ask, Advise, Assess, Assist and Arrange” [12]. Previous similar studies in China found that approximately 50% of doctors often asked about the smoking history of the patients [24, 27]. It has been reported that approximately 28–70% of doctors ask patients about their smoking status in other countries (such as Belgium, Ireland, England and Egypt) [22, 23]. Similar to Mostafa’s finding [23], the result of the current study (85.2% of participants often asked the patients about their smoking status) was relatively satisfactory. Approximately 36% of health professionals always advised smokers to quit smoking in study conducted in 12 European countries [28]. The results of a study showed that 66% of participants advised their patients to quit smoking among obstetricians/gynecologists in Ohio [29]. In line with a previous study in China [30], approximately 67 and 30% of doctors in this study often and sometimes persuaded the patients to quit smoking, respectively. The relatively acceptable behaviors of “Ask” and “Advise” in China may be attributed to Chinese tobacco control efforts from 2006 to the present, especially tobacco control education in hospitals and universities. With increasing age or professional title, an increasing number of participants asked about the patients’ smoking history or advised the patients to quit smoking. In addition, greater proportions of doctors with graduate degrees often asked about the smoking history of patients. This situation reflected the effect of education about tobacco control in hospitals and universities to a certain degree. In the current study, only approximately 5% of participants provided assistance (prescribing smoking cessation drugs for patients), which was similar to results of previous Chinese studies [26, 31]. This proportion was lower than the average level of assistance reported by a systematic literature review (44%) [13]. Approximately 15% of participants provided smoking cessation assistance among family physicians in Suez Canal University Hospitals [9]. There are some possible explanations for this difference in assistance between doctors in China and other countries. Firstly, few patients seek help for smoking cessation in Chinese hospitals. In China, offering cigarettes was considered a traditional social courtesy and a sign of respect [10]. On private occasions, the tradition of smoking has not changed fundamentally, and smoking is still treated as an individual freedom, which may result in the fact that few patients seek help for smoking cessation. Therefore, it is regarded as “offensive” and “harmful” for doctors to actively provide smoking cessation assistance [20]. Secondly, there is no requirement for doctors to actively provide smoking cessation assistance for smokers.

In this study, “Agree” response rates among pediatricians were higher than those among doctors in other departments for the majority of the items under study, which was similar to the findings of a United States study (83% of pediatricians would ask about the family members’ smoking history) but different from the results of a Poland study (23% of pediatricians would ask about the family members’ smoking history) [32]. Due to children’s physiological characteristics, children are more susceptible to adverse effects of smoke exposure [32]. Children’s exposure to smoke will lead to a series of diseases (such as asthma, bronchitis, coughing, and pneumonia) [33]. Additionally, most couples currently have only one or two children in China, and children become more important to families. In this situation, Chinese pediatricians are required to learn more (including information about smoking and smoking-related diseases) and provide more information to parents. This may explain Chinese pediatricians’ relatively high “Agree” response rates. “Agree” response rates of surgeons were lower than those of doctors in other departments for the majority of the items under study. This corresponded with the fact that the surgery department had the highest smoking rate in China among the different medical departments [34–36]. To reduce work-related stress and improve sociability [23], more surgeons have become smokers. Smoking doctors may pay less attention to knowledge of smoking, ignore the harms from smoking and be less likely to provide smoking cessation assistance [10, 23, 37].

There were also some limitations in this study. Firstly, participants were not completely randomly sampled. To enroll participants conveniently and improve the feasibility of the study, purposive sampling was used to choose the sample hospital at the third step of sampling. This recruitment method may result in selection bias. We will use complete sampling to identify participants in further studies to overcome this limitation. Secondly, our questionnaire included the “Ask”, “Advise” and “Assistance” of 5As method but did not include “Assess” or “Arrange”. Data from preinvestigation revealed that very few doctors provided “Assess” and “Arrange”. For the sake of improving the feasibility of the study, questions about “Assess” and “Arrange” were not included. Therefore, it was difficult for the study to comprehensively evaluate doctors’ practices regarding smoking control. Thirdly, the sample under study was not compared with the doctor population of China. It was unknown whether the demographic characteristics of the sample under study could precisely represent those of the doctor population of China. Therefore, care should be taken when generalizing the conclusions.

## Conclusions

In conclusion, this study revealed that doctors in Shandong Province did not have sufficient knowledge of smoking control. Slightly more than half of doctors thought that providing smoking cessation assistance was their responsibility. Only a few doctors had prescribed smoking cessation drugs for their patients.

## Data Availability

The datasets used and/or analyzed during the current study are available from the corresponding author on reasonable request (chpwang@163.com).
